# Post-Traumatic Stress Symptoms, Occupational Stress, Emotional Exhaustion, and Quiet Quitting in Workplace-Violence-Exposed Turkish Prehospital Emergency Workers: A Cross-Sectional Study

**DOI:** 10.3390/healthcare14152244

**Published:** 2026-07-23

**Authors:** Mehmet Tatlı, Ramazan Güven, Habip Balsak, Mehmet Özel, İsmail Altıntop

**Affiliations:** 1 Department of Emergency Medicine, Van Training and Research Hospital, University of Health Sciences, Van 65100, Türkiye; mehmet.tatli@sbu.edu.tr; 2 Department of Emergency Medicine, Faculty of Medicine, Istanbul Atlas University, Istanbul 34403, Türkiye; 3 Department of Midwifery, Faculty of Health Sciences, Batman University, Batman 72100, Türkiye; habip.balsak@batman.edu.tr; 4 Department of Emergency Medicine, Diyarbakır Gazi Yaşargil Training and Research Hospital, University of Health Sciences, Diyarbakır 21070, Türkiye; drmehmetozel@yahoo.com.tr; 5 Department of Emergency Medicine, Kayseri City Training and Research Hospital, University of Health Sciences, Kayseri 38080, Türkiye; ismail.altintop@sbu.edu.tr

**Keywords:** prehospital emergency medical services, ambulance personnel, workplace violence, occupational stress, post-traumatic stress disorder, emotional exhaustion, quiet quitting, Türkiye

## Abstract

(1) Background: Prehospital emergency workers frequently experience workplace violence and trauma, raising their risk of post-traumatic stress disorder (PTSD) symptoms and burnout, yet their link to quiet quitting remains poorly understood. We tested whether the PTSD–quiet quitting association is statistically patterned through perceived occupational stress and emotional exhaustion. (2) Methods: In a cross-sectional online survey, prehospital emergency professionals in Türkiye who reported workplace violence within the previous six months completed the PTSD-Short Scale, Perceived Occupational Stress Scale, Job-Related Emotional Exhaustion Scale, and Quiet Quitting Scale. Serial mediation was tested with PROCESS Model 6 (5000 bootstrap resamples), with an age- and gender-adjusted sensitivity analysis, and reporting followed the STROBE checklist. (3) Results: Of 327 respondents, 305 were analysed. PTSD symptoms were associated with quiet quitting (β = 0.460, *p* < 0.001). With both mediators entered, the direct path was non-significant (β = 0.105, *p* = 0.071), indicating full statistical mediation. The serial route through stress and exhaustion was the largest indirect pathway (B = 0.155, 95% CI [0.091, 0.217]), and the model explained 42.1% of the variance. Self-reported, single-session data mean the indirect effects are best treated as upper-bound estimates. (4) Conclusions: PTSD symptoms were indirectly related to quiet quitting through occupational stress and emotional exhaustion. The high correlation between the two mediators suggests one broad strain process rather than two distinct stages. Because the design is cross-sectional, it precludes causal ordering; longitudinal studies are needed. Clinically, the findings highlight reducing workplace violence and monitoring stress and exhaustion to limit disengagement among prehospital staff.

## 1. Introduction

Professionals working in prehospital emergency medical services (EMS) operate in one of the most psychologically demanding occupational environments. Their daily work involves routine encounters with traumatic incidents, exposure to workplace violence, and constant pressure to make rapid clinical decisions [1,2]. This combination of stressors places them at a heightened risk of adverse mental health outcomes, among which post-traumatic stress disorder (PTSD) is one of the most common. Systematic reviews and meta-analyses have estimated the prevalence of PTSD among ambulance personnel to be approximately 11–30%, substantially higher than that in the general population [1,3,4].

Workplace violence (WPV), encompassing physical and verbal aggression from patients and the public, is a widespread and well-documented occupational hazard for EMS professionals [5,6]. Empirical evidence indicates that WPV is associated with elevated PTSD symptoms, burnout, and reduced job satisfaction [7,8,9]. The persistent threat of violence may also contribute to a working atmosphere of hypervigilance and fear, compounding the stress inherent in emergency duties. In Türkiye, recent evidence from prehospital EMS personnel has documented that a substantial proportion of them reported physical violence from patients during their career (40.1%), underscoring the scope of this occupational hazard [7]. Burnout, the chronic state of emotional exhaustion, depersonalisation, and reduced personal accomplishment that develops under sustained occupational stress, is a particular concern for caregiving professionals and reaches especially high rates in emergency medicine, where it has been reported to exceed 70% in some settings and is driven by both organisational factors (workload, work–life conflict) and individual coping resources [10]. This phenomenon is not confined to the emergency department; comparably elevated exhaustion has been documented in other high-demand caregiving environments, including nurses working in correctional facilities [11], underscoring that chronic emotional labour rather than any single clinical setting is the common driver. Recognition of this burden has prompted growing interest in resilience-oriented responses, and a Cochrane review of randomised trials indicates that psychological interventions can foster resilience and modestly reduce stress and exhaustion among healthcare professionals [12]. These observations motivate closer attention to the strain processes that may link trauma exposure to behavioural withdrawal in prehospital workers.

In recent years, the term “quiet quitting” has emerged in both academic and popular discourse to describe a pattern of reduced discretionary effort in which employees consciously limit their work to the minimum requirements of their role [13,14]. Although the term is new, the phenomenon overlaps with established constructs of psychological withdrawal and has been widely interpreted in the nursing and occupational health literature as a self-protective response to chronic job stress and burnout [15,16,17,18]. In healthcare settings, recent empirical studies have identified burnout as a proximal correlate of quiet quitting tendencies [15,16,17,18,19]. In Türkiye’s centrally administered public-sector healthcare system, where formal resignation is procedurally and financially constrained, quiet quitting may operate less as a generational lifestyle choice and more as a constrained behavioural response when physical exit from the role is structurally restricted.

**Figure 1:**
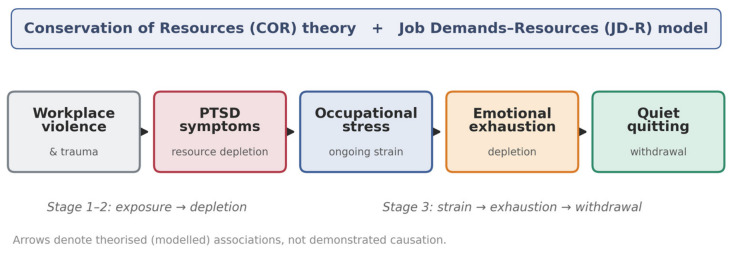
Theoretical framework integrating the Conservation of Resources (COR) theory and the Job Demands–Resources (JD-R) model. Stage 1: Resource-draining events (workplace violence, trauma). Stage 2: PTSD symptoms as severe resource depletion. Stage 3: Heightened demand vulnerability, occupational stress, emotional exhaustion, and resource-conserving withdrawal (quiet quitting).

This study draws on two complementary theoretical frameworks. The conservation of resources (COR) theory [20,21] proposes that psychological stress arises when individuals face actual or threatened loss of personal resources and that failure to replenish depleted resources can trigger a loss spiral leading to burnout. The Job Demands–Resources (JD-R) model [22] proposes that a chronic imbalance between job demands and available resources is associated with strain and exhaustion. Within these frameworks, PTSD symptoms can be conceptualised as a marker of substantial resource depletion, occupational stress as an index of ongoing strain, emotional exhaustion as a state of profound depletion, and quiet quitting as a resource-conserving pattern of reduced discretionary effort. These constructs are integrated into a single theoretical framework, which, together with the study hypotheses, is presented at the beginning of the Materials and Methods section (Figure 1).

To our knowledge, no prior study has tested a serial mediation model of the JD-R health-impairment pathway linking recent workplace violence to quiet quitting in prehospital emergency staff. The primary objective was to test whether the association between PTSD symptoms and quiet quitting is serially mediated by perceived occupational stress and job-related emotional exhaustion. The secondary objectives were to quantify the relative contributions of the single-mediator and serial pathways and to compare the serial model with simpler single-mediator alternatives in explained variance. The study contributes the first examination of this model in a Turkish prehospital emergency sample defined by recent workplace violence exposure, operationalising quiet quitting, rather than turnover intention, as the withdrawal outcome of interest.

## 2. Materials and Methods

This study was grounded in an integrated theoretical framework combining the Conservation of Resources theory and the Job Demands–Resources model. The framework (Figure 1) specifies the constructs and the directional pattern of associations evaluated in the analysis, and the formal hypotheses derived from it are stated below.

Rather than proposing a novel mechanism, this framework operationalises the well-established health-impairment pathway of the JD-R model, in which sustained job demands deplete energetic resources, precipitate strain, and culminate in behavioural withdrawal, and integrates it with the loss-spiral logic of Conservation of Resources theory; each hypothesis is derived from this pathway and is consistent with recent empirical serial-mediation studies in caregiving occupations. Workplace violence and repeated trauma exposure constitute chronic job demands and resource-draining events, so PTSD symptoms are conceptualised as a marker of severe resource depletion that is positively associated with quiet quitting, a resource-conserving reduction in discretionary effort (H1); this proposition is consistent with recent evidence linking post-traumatic stress and violence exposure to disengagement and turnover-related outcomes in healthcare workers [23,24,25].

In line with the appraisal stage of the JD-R demand–strain sequence, perceived occupational stress is expected to mediate the association between PTSD symptoms and quiet quitting (H2), reflecting the ongoing strain generated when depleted workers continue to face high demands [26,27].

Consistent with the depletion stage of the same pathway, job-related emotional exhaustion, the core energetic component of burnout, is expected to mediate this association (H3); a growing body of empirical work identifies emotional exhaustion as the proximal correlate of withdrawal and reduced discretionary effort, including quiet quitting, in nurses and emergency personnel [17,28,29,30,31].

Because Conservation of Resources theory frames strain and exhaustion as sequential stages of a loss spiral rather than independent states, perceived occupational stress and emotional exhaustion are expected to operate as serial mediators, such that PTSD symptoms are associated with quiet quitting through a demand–appraisal-to-depletion sequence (H4); comparable serial mediation chains, in which a workplace stressor predicts a withdrawal outcome through stress and burnout in turn, have been demonstrated empirically in nursing and healthcare samples [25,26,27,31].

**H1.**  *Post-traumatic stress disorder (PTSD) symptoms are positively associated with quiet quitting tendencies among prehospital emergency workers exposed to recent workplace violence.*

**H2.**  *Perceived occupational stress mediates the association between PTSD symptoms and quiet quitting.*

**H3.**  *Job-related emotional exhaustion mediates the association between PTSD symptoms and quiet quitting.*

**H4.**  *Perceived occupational stress and job-related emotional exhaustion function as serial mediators of the association between post-traumatic stress disorder (PTSD) symptoms and quiet quitting.*

### 2.1. Study Design

We employed a cross-sectional, correlational research design and report our findings in accordance with the STROBE checklist for cross-sectional studies.

### 2.2. Setting and Participants

Data were collected between 25 October 2025 and 7 February 2026. The target population comprised prehospital emergency medical personnel employed in the 112 emergency ambulance service in Türkiye. The Turkish 112 prehospital workforce is multidisciplinary; emergency medical technicians and paramedics provide the majority of field care, working alongside a smaller number of physicians and nurses or midwives, together with non-clinical staff, such as ambulance drivers and call-centre or dispatch personnel. In the demographic categories used here, the “Other” group comprises mainly these ancillary roles (for example, drivers and dispatchers) rather than an additional clinical profession. Recruitment employed a convenience sampling strategy using an online, self-administered Google Forms (Google LLC, Mountain View, CA, USA) survey distributed via professional WhatsApp groups, occupational email lists, and informal peer networks of prehospital emergency professionals in Türkiye. The survey link circulated organically across regions and reached personnel from multiple provinces. No quota was set for the region of employment, and the resulting sample was therefore drawn from across Türkiye rather than restricted to the principal investigator’s province. Distribution through line management or human resource channels was deliberately avoided to minimise perceived coercion. Eligibility criteria were: (i) self-reported workplace violence exposure within the preceding six months, assessed by a single screening item (“Within the past six months, have you been exposed to verbal aggression, physical aggression, or sexual harassment at your workplace from patients, patients’ relatives, or colleagues?”); (ii) active employment in prehospital emergency services for at least six months; (iii) age 18 years or older; and (iv) provision of voluntary electronic informed consent. Pre-existing psychiatric diagnoses were not used as exclusion criteria and were not systematically assessed in this study. The province of employment was not collected. Because the survey circulated organically through peer networks, the total number of personnel who received the link could not be determined; therefore, a formal response rate could not be computed. This is acknowledged as a limitation. The required sample size was determined using G*Power 3.1 (Heinrich Heine University Düsseldorf, Düsseldorf, Germany) [32], which indicated that a minimum of 77 participants was required for multiple regression analysis with a medium effect size (f^2^ = 0.15), α = 0.05, and power = 0.80. Our final analytic sample of *n* = 305 substantially exceeded this requirement. Following Fritz and MacKinnon’s [33] simulation tables, a sample of approximately 250 yields ≥80% power to detect indirect effects of the magnitude observed in our serial pathway (β = 0.197), supporting adequate statistical power for the bootstrap mediation analysis.

### 2.3. Ethical Considerations

This study was conducted in accordance with the principles of the Declaration of Helsinki. Ethical approval was obtained from the Non-Invasive Clinical Research Ethics Committee of the University of Health Sciences, Van Training and Research Hospital (decision number GOKAEK/2025-08-03, dated 24 October 2025), which is affiliated with the principal investigator. Because the study involved an anonymous, voluntary online survey of a competent adult professional population without identifiable data collection, single-institution ethical approval was consistent with national regulations governing minimal-risk anonymous survey research. Data collection commenced only after ethical approval was granted. Participants provided electronic informed consent before accessing the survey items. Participation was anonymous, and no personally identifiable information (including IP address or email) was collected. All data were stored on a password-protected institutional drive that was accessible only to the research team.

### 2.4. Measures

Four validated self-report instruments with established Turkish-language adaptations were used in this study. The internal consistency was re-estimated in the present sample.

#### 2.4.1. Post-Traumatic Stress Disorder-Short Scale (PTSD-SS)

The PTSD-SS is a brief self-report screening instrument for PTSD symptomatology over the past week, derived from the National Stressful Events Survey PTSD Short Scale developed by LeBeau et al. [34], consistent with the broader DSM-based primary-care PTSD screening tradition [35,36]. Participants responded with reference to their most recent experience of workplace violence. The Turkish adaptation by Evren et al. [37] reported a Cronbach’s α of 0.87. Items are rated on a 5-point Likert scale (1–5); total scores range from 9 to 45, with higher scores indicating a greater probability of PTSD. In the present sample, Cronbach’s α was 0.89.

#### 2.4.2. Perceived Occupational Stress Scale (POSS)

Developed by Marcatto et al. [38], the POSS is a four-item, 5-point Likert instrument measuring perceived job-related stress; total scores range from 4 to 20, with higher values indicating greater stress. The Turkish adaptation was performed by Yıldırım, Dilekçi, Marcatto, and Gómez-Salgado [39], who reported a Cronbach’s α of 0.85 in their Turkish sample. In the original validation, the authors reported α = 0.82 [38]. In the present sample, Cronbach’s α was 0.85.

#### 2.4.3. Job-Related Emotional Exhaustion Scale (JREES)

The JREES, originally used by Wharton [40] in her study on emotional labour in service work, is a unidimensional six-item instrument that assesses emotional exhaustion at work. The Turkish adaptation was performed by Günay [41], whose exploratory factor analysis confirmed a single-factor structure explaining approximately 68% of the total variance, with a Cronbach’s α of 0.903. Items are rated on a 5-point Likert scale (0 = “I never feel this way at work” to 4 = “I feel this way every day”), yielding total scores ranging from 0 to 24. Higher scores indicate more severe exhaustion. In the present sample, Cronbach’s α was 0.93.

#### 2.4.4. Quiet Quitting Scale (QQS)

The QQS used in this study comprised seven items measuring the quiet quitting construct. The original measurement work by Anand, Doll, and Ray [13] developed and validated a two-factor instrument assessing both quiet quitting (employee-level disengagement) and quiet firing (managerial-level devaluation); the seven-item, unidimensional quiet-quitting structure used here corresponds to the employee-disengagement subscale. The Turkish-language adaptation by Yıldız, Ekingen, Gün, and Yıldırım [42] examined the seven-item employee disengagement subscale in a Turkish healthcare worker sample and reported that confirmatory factor analysis supported a unidimensional structure with an acceptable fit and a Cronbach’s α of 0.81. Items are rated on a 5-point Likert scale (1–5), and total scores range from 7 to 35, with higher scores reflecting a greater propensity for quiet quitting. In the present sample, Cronbach’s α was 0.78, slightly below the conventional 0.80 benchmark but within the range previously reported for this scale in healthcare populations [42]. This lower internal consistency is acknowledged as a limitation.

### 2.5. Statistical Analysis

All analyses were performed using IBM SPSS Statistics version 24.0 (IBM Corp., Armonk, NY, USA) and the PROCESS macro version 4.2 for SPSS (Model 6) developed by Hayes [43]. Descriptive statistics, Pearson and Spearman bivariate correlations, and internal consistency estimates were computed using SPSS. Multicollinearity among the predictors was assessed using variance inflation factors (VIF) and tolerance values. Common method variance was screened with Harman’s single-factor test via principal component analysis as an initial diagnostic [44]; because the survey did not include a pre-specified, theoretically unrelated marker variable, formal control of common method variance was limited to this test. In addition, the four-factor measurement model was examined by confirmatory factor analysis using the semopy package (version 2.3) in Python (version 3.10); construct reliability and convergent validity were summarised with composite reliability and average variance extracted, discriminant validity was assessed with the Fornell–Larcker criterion and the heterotrait–monotrait (HTMT) ratio [45,46], and model fit was judged against conventional cutoffs (CFI ≥ 0.90, RMSEA ≤ 0.08) [47]; the serial mediation model was additionally re-estimated as a latent-variable structural equation model. For the serial mediation analysis, PROCESS Model 6 was used with 5000 bias-corrected bootstrap resamples to estimate the total, direct, and specific indirect effects with 95% confidence intervals. Effect sizes (R^2^ and Cohen’s f^2^) were computed for each regression step following Cohen’s [48] benchmarks (small = 0.02, medium = 0.15, large = 0.35). Supplementary model comparison analyses contrasted the full serial model with single-mediator alternatives. To assess the robustness of the indirect effects of demographic confounding, PROCESS Model 6 was re-run with age and gender entered as covariates, and the adjusted estimates are reported in Supplementary Table S1. Because the design is cross-sectional, indirect pathways are interpreted as theoretically motivated statistical patterns of association rather than as tests of causal or temporal ordering; cross-sectional mediation estimates can be biased relative to longitudinal mechanisms [49].

## 3. Results

### 3.1. Participant Flow and Sample Characteristics

**Figure 2:**
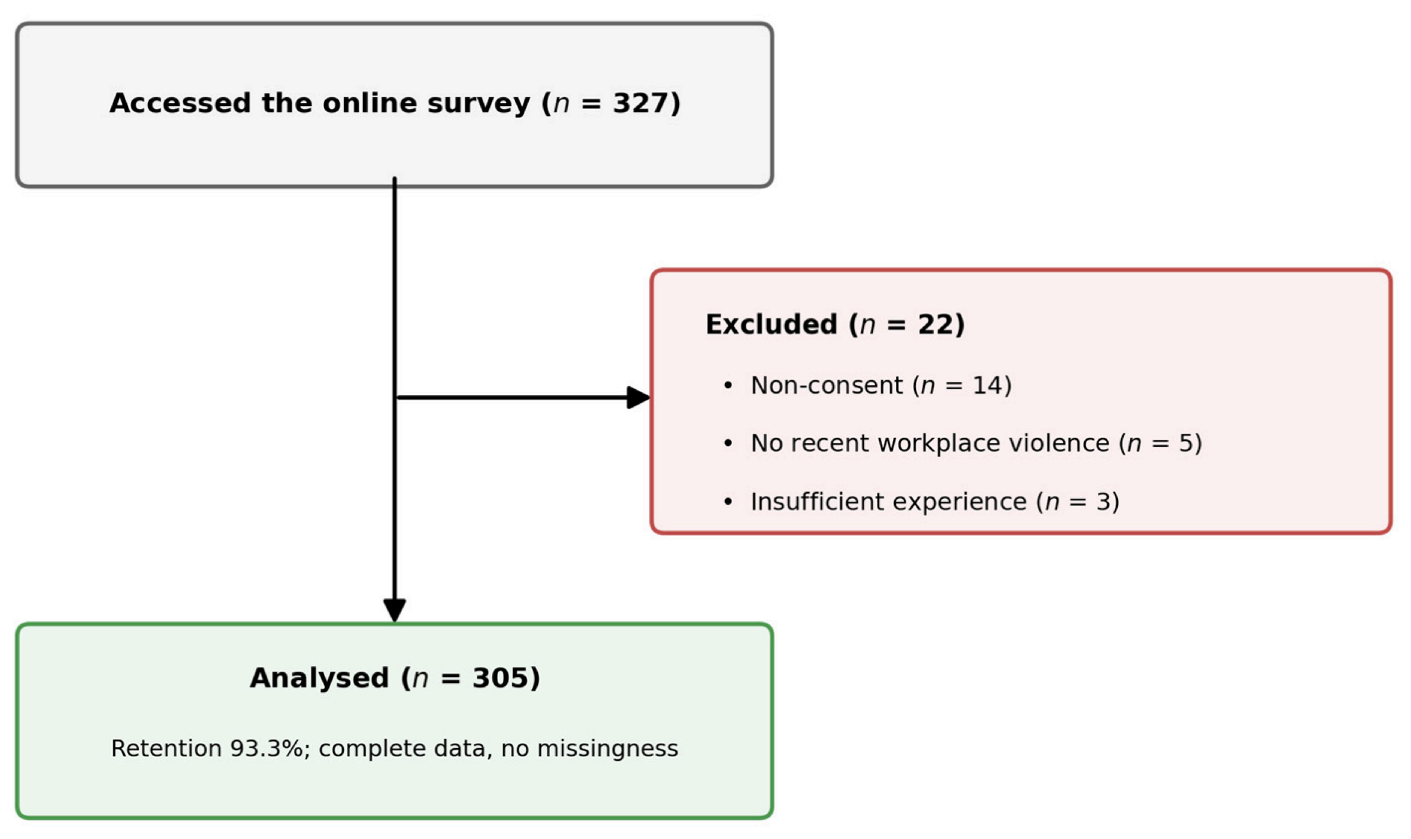
STROBE participant flowchart.

Of the 327 individuals who accessed the survey, 14 were excluded for non-consent, 5 for absence of recent workplace violence exposure, and 3 for insufficient experience, yielding a final analytic sample of *n* = 305 (retention 93.3%). All retained respondents provided complete data on all four study instruments, with no item-level missingness. The participant flow is illustrated in Figure 2.

As shown in Table 1, the sample was predominantly male (*n* = 167, 54.8%), aged 26–35 years (*n* = 189, 62.0%), married (*n* = 215, 70.5%), university-educated (*n* = 259, 84.9%), and comprised EMTs/paramedics (*n* = 241, 79.1%). Most of them worked rotating shifts (*n* = 252, 82.6%).

### 3.2. Common Method Variance

Because all variables were measured through self-report instruments in the same session, common method variance was assessed. Harman’s single-factor test indicated that the first unrotated component accounted for 39.7% of the total variance, below the conventional 50% threshold [44], which provided initial reassurance.

### 3.3. Descriptive Statistics and Bivariate Correlations

Table 2 presents the descriptive statistics, reliability estimates, and correlation matrix for the primary study variables. All scales demonstrated acceptable to excellent internal consistency (α = 0.78–0.93). All four variables were significantly and positively intercorrelated (all *p* < 0.001), with coefficients ranging from moderate (Pearson r = 0.46/Spearman ρ = 0.46, PTSD and quiet quitting) to strong (Pearson r = 0.81/Spearman ρ = 0.83, perceived stress and emotional exhaustion). The variance inflation factors did not exceed 3.32, and the tolerance values exceeded 0.30, indicating multicollinearity within conventionally acceptable limits, although the strong stress–exhaustion association is discussed as a limitation.

### 3.4. Serial Mediation Analysis

**Figure 3:**
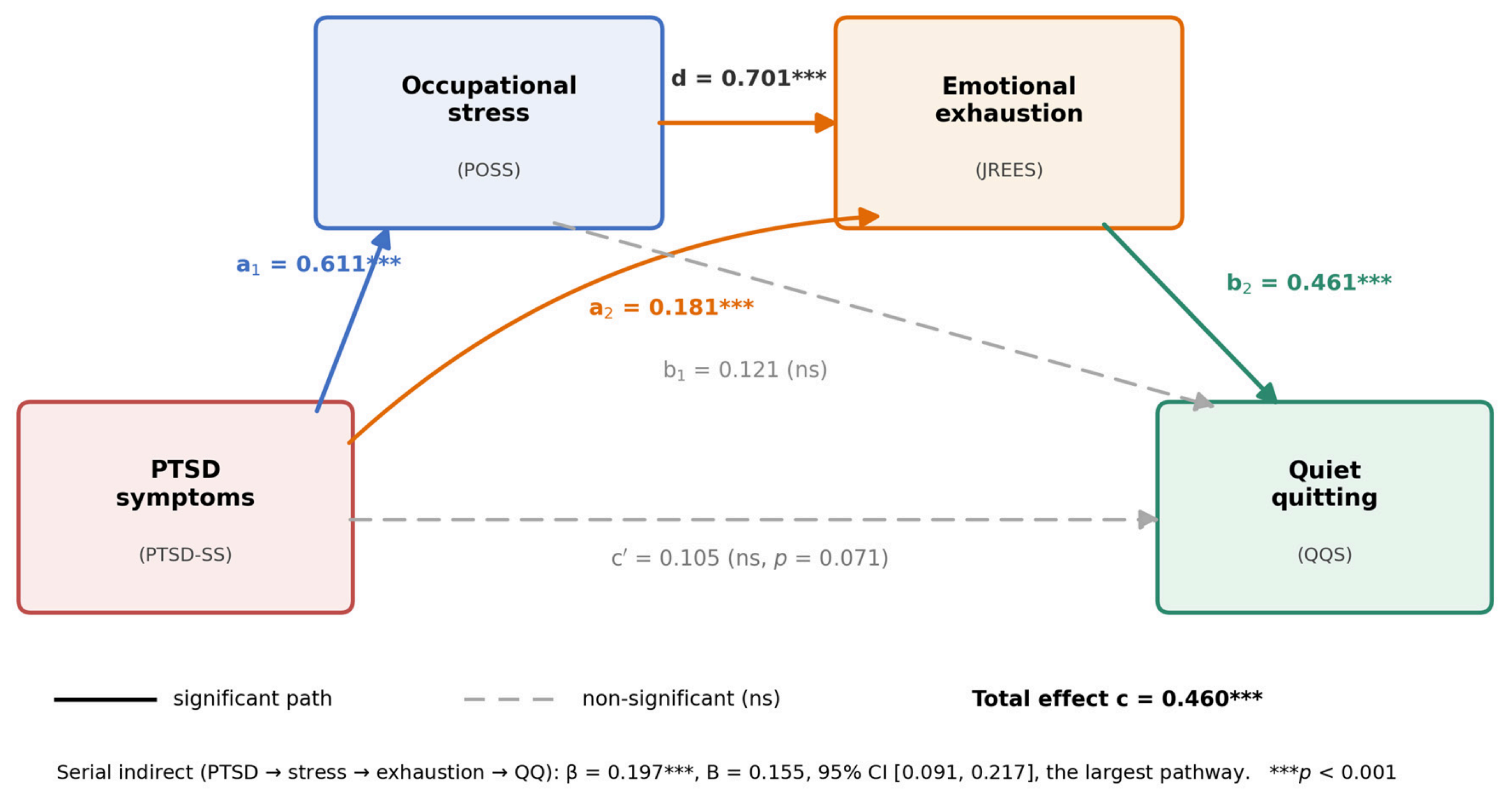
Serial mediation model with standardised path coefficients. PTSD symptoms (PTSD-SS) → perceived occupational stress (POSS) → job-related emotional exhaustion (JREES) → quiet quitting (QQS). Solid lines indicate significant paths; dashed lines indicate non-significant paths. Total effect c = 0.460 ***; direct effect c′ = 0.105 (ns, *p* = 0.071), consistent with the full statistical mediation (Figure 3). The serial indirect pathway (β = 0.197 ***; B = 0.155, 95% CI [0.091, 0.217]) was the largest indirect route. *** *p* < 0.001; ns = not significant.

The results of the serial mediation analysis (PROCESS Model 6) are presented in Table 3 and illustrated in Figure 3. The total association of PTSD symptoms with quiet quitting was statistically significant (B = 0.361, SE = 0.040, β = 0.460, 95% CI [0.282, 0.440], *p* < 0.001), supporting H1. After both mediators were entered, the direct path was reduced to non-significance (B = 0.082, SE = 0.045, β = 0.105, 95% CI [−0.007, 0.172], *p* = 0.071), consistent with a fully mediated statistical pattern.

**Figure 4:**
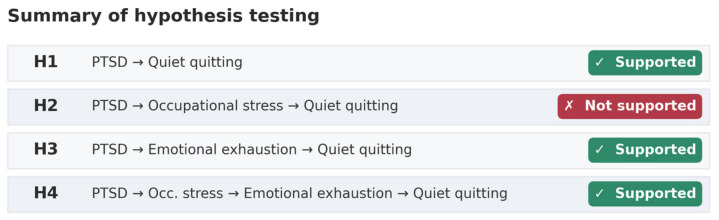
Summary of the hypothesis testing results. H1, H3, and H4 were supported, while H2 (single mediator pathway through perceived occupational stress alone) was not supported.

The total indirect effect was statistically significant (B = 0.279; 95% CI [0.199, 0.364]). The examination of specific indirect pathways yielded mixed support for the individual mediation hypotheses. The single-mediator pathway through perceived stress alone was not significant (B = 0.058, 95% CI [−0.006, 0.134]), and H2 was therefore not supported. Although the bivariate correlation between perceived stress and quiet quitting was moderate (r = 0.577), this association was substantially attenuated after emotional exhaustion was included in the regression model, consistent with the suppression of the unique stress-to-quitting path by the stronger stress–exhaustion–quitting pattern and the high shared variance between the two mediators. Surprisingly, the magnitude of the single-mediator pathway through emotional exhaustion exceeded what bivariate correlations alone suggested and was statistically significant (B = 0.065, 95% CI [0.029, 0.113]); thus, H3 was supported. The complete serial pathway from PTSD symptoms via perceived stress and emotional exhaustion to quiet quitting was the largest indirect route (B = 0.155, 95% CI [0.091, 0.217]), supporting H4. The hypothesis outcomes are summarised in Figure 4. Because the design was cross-sectional, this serial pattern was interpreted as a statistical association rather than evidence for a temporal or causal sequence. To assess robustness against demographic confounding, PROCESS Model 6 was re-estimated with age and gender entered as covariates (Supplementary Table S1). The covariate-adjusted estimates were materially unchanged: the indirect effect through emotional exhaustion (adjusted B = 0.066, 95% CI [0.029, 0.113]) and the serial pathway through stress and exhaustion (adjusted B = 0.153, 95% CI [0.095, 0.214]) remained statistically significant, whereas the single-mediator pathway through stress alone remained non-significant (adjusted B = 0.051, 95% CI [−0.013, 0.128]). A full collinearity assessment confirmed that the variance inflation factors remained within the conventionally acceptable limits, indicating no problematic multicollinearity among the predictors.

### 3.5. Effect Sizes

The model accounted for the substantial variance at each step. For perceived stress, R^2^ = 0.370 (Cohen’s f^2^ = 0.587, large). For emotional exhaustion, R^2^ = 0.698 (Cohen’s f^2^ = 2.311, large). For quiet quitting, R^2^ = 0.421 (Cohen’s f^2^ = 0.727, large). All values exceeded Cohen’s [48] large-effect benchmark of 0.35.

### 3.6. Model Comparison

The serial model (R^2^ = 0.421) accounted for greater variance than the burnout-only (R^2^ = 0.395; ΔR^2^ = 0.026, *p* < 0.05) and stress-only (R^2^ = 0.341; ΔR^2^ = 0.080, *p* < 0.001) alternatives. All three models yielded large effect sizes. The results are presented in Table 4.

### 3.7. Measurement Model, Discriminant Validity, and Latent-Variable Robustness

The four-factor measurement model (PTSD symptoms, perceived occupational stress, emotional exhaustion, and quiet quitting) showed acceptable fit, χ^2^(293) = 618.4, CFI = 0.930, TLI = 0.922, RMSEA = 0.060 (full standardised loadings, reliability, and discriminant-validity statistics are provided in Supplementary Tables S3 and S4). Standardised loadings were satisfactory for PTSD symptoms (0.57–0.80), perceived occupational stress (0.59–0.83), and emotional exhaustion (0.70–0.91), and more variable for quiet quitting (0.30–0.80); composite reliability was adequate for all constructs (CR = 0.77–0.93), whereas the average variance extracted for quiet quitting was low (AVE = 0.35), consistent with the modest internal consistency of that scale. Discriminant validity was supported for most construct pairs but was limited between perceived occupational stress and emotional exhaustion: their HTMT ratio was 0.90 and their latent (disattenuated) correlation was 0.91, and the Fornell–Larcker criterion was not met for this pair. Nevertheless, a two-factor solution for these ten items fit significantly better than a one-factor ‘strain’ solution, Δχ^2^(1) = 52.8, *p* < 0.001 (two-factor CFI = 0.945 versus one-factor CFI = 0.924), indicating that the two constructs are statistically separable. Re-estimating the serial model as a latent-variable structural equation model reproduced the manifest results (CFI = 0.930, RMSEA = 0.060), with emotional exhaustion as the proximal predictor of quiet quitting (β = 0.55) and only a weak unique perceived-stress path (β = 0.15); an alternative model collapsing the two mediators into a single latent strain factor fit slightly less well (CFI = 0.917). Together, these analyses indicate that perceived occupational stress and emotional exhaustion are statistically distinguishable but strongly overlapping facets of a common strain process, and that the mediation pattern is not an artefact of measurement unreliability.

## 4. Discussion

In this cross-sectional convenience sample of 305 prehospital emergency workers with recent workplace violence exposure, the association between PTSD symptoms and quiet quitting was not direct but was fully statistically mediated by perceived occupational stress and emotional exhaustion. The serial indirect pathway from PTSD symptoms through stress and exhaustion to quiet quitting was the largest of the three indirect routes tested (β = 0.197), and the full model accounted for 42.1% of the variance in quiet quitting (Cohen’s f^2^ = 0.727, a large effect according to conventional benchmarks). These results have implications for understanding the patterns of psychological strain and behavioural withdrawal in trauma-exposed healthcare populations, although the cross-sectional design does not permit conclusions about temporal ordering or causation.

### 4.1. Principal Findings: Emotional Exhaustion as the Primary Mediator of the PTSD–Quiet Quitting Association

The significant positive association between PTSD symptoms and quiet quitting (H1 supported; β = 0.460, *p* < 0.001) extends the established literature on PTSD and adverse occupational outcomes in EMS [1,3,4] by linking trauma-related symptomatology to contemporary indicators of disengagement. This finding is consistent with recent evidence that post-traumatic stress symptoms predict withdrawal-related outcomes in healthcare workers: in a large sample of nurses, PTSD symptoms were associated with turnover intention both directly and indirectly through presenteeism [23], and post-traumatic stress has been shown to exert an indirect effect on turnover intention via burnout in path-analytic models [24]. Comparable co-occurrence of PTSD severity with the exhaustion and disengagement components of burnout has been documented among intensive care staff [50]. The total association corresponded to approximately 21% of the variance in quiet quitting accounted for by PTSD symptoms alone. Once stress and exhaustion were entered into the model, the direct path was reduced to non-significance, indicating that the PTSD–quiet quitting association is indirect rather than direct.

The single-mediator pathway through perceived stress alone (H2) was not statistically significant (B = 0.058, 95% CI [−0.006, 0.134]). This does not imply that stress is irrelevant; the bivariate correlation (r = 0.577) was significant. However, once exhaustion was controlled, the unique contribution of perceived stress was attenuated. Perceived stress appears to be associated with quiet quitting primarily because it co-occurs with exhaustion. This pattern is consistent with Hobfoll’s [20] resource loss spiral and the JD-R model’s [22] distinction between acute strain and chronic exhaustion. A similar pattern was reported by Galanis et al. [51], who found that workplace bullying was associated with quiet quitting among Greek nurses through coping strategies.

The exhaustion pathway (H3) was statistically significant (B = 0.065, 95% CI [0.029, 0.113]), supporting emotional exhaustion as a proximal correlate of quiet quitting tendencies. This finding is consistent with recent studies on nursing populations. Gün, Balsak, and Ayhan [15] reported that burnout mediates the association between organisational support and quiet quitting among Turkish nurses. Building on this, Gün, Çetinkaya Kutun and Söyük [17] showed that turnover intention partially mediated the burnout–quiet quitting relationship, and a two-mediator serial model in Korean nurses showed that missed nursing care and job satisfaction sequentially linked presenteeism to turnover intention [18], illustrating that multi-stage serial mediation pathways are well-suited to modelling withdrawal-related outcomes in nursing. Galanis et al. [52] further demonstrated that moral resilience was associated with reduced quiet quitting and burnout among Greek nurses. Our findings extend the recent national-scale evidence by Ekşi et al. [7], who documented high rates of violence exposure among Turkish prehospital EMS personnel (*n* = 501; 40.1% reporting physical violence over their career), by demonstrating that the resulting exhaustion may itself function as a behavioural correlate of quiet quitting tendencies in workers carrying PTSD symptom load. Taken together, these findings suggest that exhaustion represents a behavioural threshold at which remaining resources are increasingly redirected from discretionary effort towards self-preservation. Critically, however, our findings diverge from parts of this literature in two respects: whereas several nursing studies treat perceived stress as an independent predictor of withdrawal, the unique contribution of perceived stress here was suppressed once emotional exhaustion was modelled, and our outcome was enacted quiet quitting rather than turnover intention, indicating that behavioural withdrawal in prehospital staff may be governed more by depletion than by the appraisal of stress alone.

The serial pathway (H4; B = 0.155, 95% CI [0.091, 0.217], β = 0.197) was the largest indirect route. This pattern is theoretically consistent with both COR theory [20,21] and the JD-R health-impairment process [22]: PTSD symptoms can be conceptualised as a correlate of severe resource depletion, which is in turn statistically associated with higher demand appraisal, greater exhaustion, and a resource-conserving pattern of reduced discretionary effort. Importantly, this pattern complements rather than duplicates earlier nursing mediation studies, which have tested different predictor–mediator configurations [15,17,18,51]. The serial pattern reported here represents a contribution from the prehospital EMS context, with PTSD symptoms as the upstream variable. Because the data are cross-sectional, alternative orderings of the same variables are statistically plausible and cannot be ruled out. For example, exhaustion might precede or shape the appraisal of stress; quiet quitting tendencies might themselves feed back into perceptions of stress and exhaustion over time; or unmeasured third variables (workplace climate, prior trauma history, dispositional negative affectivity) might generate spurious associations among the four constructs. Longitudinal designs with at least three measurement occasions are required to discriminate among these possibilities [49].

### 4.2. Model Comparison and Construct Overlap

Beyond statistical fit, the comparison of nested models carries an interpretive question that directly affects how these findings should be used. The serial model improved on the exhaustion-only model by only a small margin (ΔR^2^ = 0.026; full values in Table 4). This modest increment suggests that, for the practical purpose of explaining the variance in quiet quitting, most of the predictive work is done by emotional exhaustion as the proximal correlate; adding perceived stress as a prior, distinct stage buys relatively little additional explanatory power. The serial two-mediator structure is therefore best justified on theoretical rather than purely empirical grounds: it is supported by the COR and JD-R frameworks that motivated the study, but the data alone would not compel a two-stage model over a simpler one-mediator model.

This reading is reinforced by the very high correlation between the two mediators, expected because emotional exhaustion is commonly (Pearson r = 0.81, ~66% shared variance [SV]; Spearman ρ = 0.83). This strong association was, in one sense, theoretically conceptualised as a consequence of prolonged occupational stress rather than as a fully independent construct, and collinearity diagnostics did not indicate a problematic level. Nevertheless, a correlation of this magnitude raises a genuine discriminant-validity question: perceived occupational stress and emotional exhaustion may not be cleanly separable stages of a sequence but rather overlapping facets of a single underlying strain dimension, measured at slightly different points on an appraisal-to-depletion continuum. If that is the case, the serial pathway we report should be read less as evidence of two distinct psychological mechanisms operating in turn and more as a statistical decomposition of one broad strain process into correlated components. This interpretive caution does not undermine the central finding that the PTSD–quiet quitting association is fully indirect through these strain variables, but it does temper any claim that stress and exhaustion are functionally independent intervention targets for reducing quiet quitting. Therefore, we retained the serial model as a theoretically plausible statistical pattern rather than as evidence of a confirmed temporal sequence. Manifest-variable mediation, as used here, cannot resolve this question; latent-variable, bifactor, or experimentally manipulated designs would be required to establish whether the two constructs are genuinely separable or better modelled as one. Moreover, because all constructs were assessed by self-report in a single session, part of this shared variance, and of the large effect sizes observed, may reflect common-method variance; the mediated estimates are therefore best read as upper-bound values rather than as evidence of two functionally independent mechanisms.

The interpretive weight placed on the serial structure also depends on how cleanly quiet quitting is distinguished from the strain constructs that precede it. Recent scale-development studies have established quiet quitting as an empirically distinguishable construct, demonstrating discriminant validity against neighbouring constructs such as work disengagement, job stress, and alienation [13,53]. At the same time, conceptual analyses in healthcare emphasise that quiet quitting shares substantial variance with burnout and cynicism and is frequently described as a behavioural manifestation of exhaustion rather than a wholly separate phenomenon [54,55]. This dual position, distinct in measurement yet overlapping in aetiology, is consistent with the present pattern of results, in which emotional exhaustion carries most of the predictive burden while the incremental contribution of the second mediator remains modest.

### 4.3. The Turkish Prehospital Context: Heterogeneous Demands, Workforce Structure, and Quiet Quitting Under Constraint

Several features of the prehospital setting in Türkiye help to contextualize the pattern observed here, although the province of employment was not collected, and the following interpretations are therefore offered as plausible contexts rather than relationships tested in our data. Türkiye’s 112 emergency ambulance system serves approximately 86 million people across regions with marked geographic and operational heterogeneity. Recent survey evidence from Turkish prehospital personnel reports that a substantial proportion of them experience physical violence from patients over their career (40.1%) [7]. This high background exposure is consistent with the upstream role of workplace violence in this model. Two structural features may contribute to this load. First, Türkiye’s healthcare workforce density is among the lowest in the OECD (2.4 physicians and 2.9 nurses per 1000 population in 2023, versus OECD averages of 3.9 and 9.2) [56], which in terms of Job Demands–Resources represents elevated baseline demand across the sector [22]. Second, public-sector personnel are appointed and reassigned through a centralized civil-service placement process that can entail geographic and social dislocation [57]. Within the Conservation of Resources theory [20,21], such dislocation represents a depletion of social and instrumental resources that may add to the resource loss produced by clinical trauma. Together, high demand and constrained mobility offer one plausible explanation for why, in this sample, PTSD symptoms were associated with quiet quitting indirectly through stress and exhaustion rather than through formal exit from the role.

The interpretation of quiet quitting may also be context-dependent. In much Western discourse, quiet quitting is framed as a generational rebalancing of work and personal life [14]. The Turkish public sector differs in terms of centralized placement and reassignment [57], constrained inter-institutional mobility, and procedural and financial costs of resigning from a permanent post, which may mean that reduced discretionary effort operates less as a freely chosen lifestyle stance and more as a constrained response when physical exit is structurally restricted. This reading is speculative in the absence of region-level and longitudinal data, but it is consistent with our finding that the PTSD–quiet quitting association was carried by stress and exhaustion rather than by overt withdrawal. This implies that interventions cannot rely on individual-level mental health support alone; structural levers such as more flexible placement and transfer policies and explicit institutional acknowledgement that geographic displacement and chronic violence exposure are occupational stressors are likely to matter alongside clinical support. We return to these levers with appropriate caution regarding the strength of the underlying evidence in Section 4.5.

### 4.4. Limitations and Future Directions

This study had several limitations that qualify the interpretation of its findings. First, the cross-sectional design precludes causal or temporal inference: the serial pattern represents a set of statistical associations rather than a psychological sequence unfolding over time, and cross-sectional mediation estimates can be biased relative to the longitudinal mechanisms they are intended to approximate. The reported ordering should therefore be read as a plausible hypothesis for multi-wave testing rather than as established temporal precedence.

Second, all data were self-reported in a single online session, raising the possibility of common method variance (CMV) and social desirability bias. Harman’s single-factor test (first unrotated component 39.7%, below the conventional 50% threshold) and a full collinearity assessment were reassuring but not decisive, and, because no theoretically unrelated marker variable was included in the survey, marker-variable approaches were not feasible in this dataset. As CMV tends to inflate associations among self-reported variables, the indirect effects reported here are best treated as upper-bound estimates.

Third, the survey required respondents to disclose sensitive psychological information. Precarious anonymity within tightly networked professional communities, hierarchical concerns that honest reporting could affect professional standing, and, most importantly, the avoidance symptom cluster of PTSD, whereby the most severely affected individuals may have been unable to engage with trauma-related items, plausibly produced selection bias and systematic under-reporting. The likely direction of this bias is conservative, so the true associations may be stronger than those observed.

Fourth, several design choices constrain internal validity. Demographic and occupational variables, notably age, gender, and years of experience, were measured but not entered as covariates, so confounding of the indirect effects cannot be excluded; workplace violence was captured with a single screening item rather than a validated multidimensional instrument; and the PTSD-SS does not attribute symptoms specifically to workplace violence, so the symptoms reported may reflect other occupational or non-occupational exposures. The two mediators also shared substantial variance (r = 0.825), so whether perceived occupational stress and emotional exhaustion are separable stages or overlapping facets of a broader strain construct cannot be resolved with the manifest-variable mediation used here.

Fifth, recruitment relied on convenience sampling through professional online networks. The analytic sample (*n* = 305), although adequate for the statistical procedures used, was skewed towards emergency medical technicians and paramedics (79.1%) and towards university-educated respondents (84.9%) and cannot be considered representative of the national prehospital workforce; personnel outside professional online networks, those with the most severe avoidance-related symptoms, and those with the least discretionary time were probably under-represented, further limiting generalisability. Taken together, these limitations indicate that the present findings are best regarded as theory-consistent associations requiring confirmation, and future research should employ multi-wave longitudinal designs with pre-specified marker variables, covariate-adjusted and sensitivity analyses, latent-variable or bifactor modelling of the strain constructs, other-source and objective outcome indicators, and region-stratified or quota sampling to test the robustness and generalisability of the model.

### 4.5. Practical Implications

Interpreted within the limits of a cross-sectional design, the present findings indicate that the PTSD–quiet quitting association operated statistically through occupational stress and emotional exhaustion, suggesting that interventions targeting a single point of the pathway are unlikely to suffice. We therefore frame the implications at three levels, distinguishing recommendations that follow directly from our data from those that extend from the broader literature.

The first level concerns exposure reduction. Because workplace violence was both the eligibility criterion and the conceptual starting point of the model, primary prevention of violence is the implication that follows most directly from our data. Structured violence-prevention and de-escalation training, non-punitive incident reporting, and environmental safeguards are consistent with international evidence on violence against EMS personnel [6,9] and fall within the existing authority of regional emergency coordinators and occupational safety committees.

The second level concerns the early identification of strain. The full statistical mediation observed in our model implies that, by the time reduced discretionary effort becomes visible, stress and exhaustion processes may already be well established. Routine, confidential psychological screening integrated into occupational health surveillance, together with structured peer-support arrangements after critical incidents, targets this intermediate stage of the pathway and is consistent with evidence on burnout and resilience in emergency medicine personnel [10].

The third level concerns access to specialist care and system-level policy. Personnel with established symptoms require timely access to evidence-based, trauma-focused psychotherapy, and meta-analytic evidence indicates that psychological interventions can foster resilience and modestly reduce stress and exhaustion among healthcare professionals [12]. At the governance level, mandating minimum standards for mental health support in prehospital services, and recognising the structural employment constraints of the Turkish public-sector context [57], extend beyond our data but are consistent with the literature on quiet quitting and workforce sustainability in health systems [51,52]. These governance-level suggestions are illustrative extensions of the broader literature rather than direct implications of the present cross-sectional findings.

We emphasise that the first level follows most directly from the study design, whereas the second and third levels combine our findings with external evidence. The effects of such interventions on workforce-level outcomes, including absenteeism, turnover, and patient-safety indicators, remain to be established in implementation research.

## 5. Conclusions

In this cross-sectional convenience sample of Turkish prehospital emergency personnel with recent workplace violence exposure, PTSD symptoms and quiet quitting were associated at the bivariate level, an association that was fully attenuated to non-significance once perceived occupational stress and emotional exhaustion were included in the model. The serial route through stress and exhaustion was the largest indirect pathway. Given the high correlation between the two mediators (Pearson r = 0.81; HTMT = 0.90) and the small incremental gain of the serial model over the exhaustion-only model, this pattern is best interpreted as the statistical decomposition of a broad strain dimension rather than as evidence for two temporally discrete stages. These findings converge with recent mediation research in nursing populations [15,17,18,52] and extend it to the prehospital EMS context with PTSD symptoms as the upstream variable of interest. Because the design is cross-sectional, no conclusions regarding temporal or causal ordering are warranted. Within these limits, the results are consistent with integrated organisational strategies that address trauma-related symptoms, occupational stressors, and exhaustion together, and with treating mental health support for prehospital workers as a systemic rather than discretionary concern. Longitudinal research and intervention studies are needed to test temporal ordering and whether modifying one element of this pattern is associated with changes in the others.

## Figures and Tables

**Table 1:** Participant demographics (*n* = 305).

Variable	Category	*n*	%
Gender	Male	167	54.8
	Female	138	45.2
Age (years)	18–25	23	7.5
	26–35	189	62.0
	36–45	75	24.6
	46–55	9	3.0
	56 or older	9	3.0
Marital status	Married	215	70.5
	Single	90	29.5
Education	University	259	84.9
	Postgraduate	46	15.1
Profession	Physician	15	4.9
	Nurse/Midwife/Other clinical	21	6.9
	EMT/Paramedic	241	79.1
	Other	28	9.2
Experience (years)	5 or fewer	68	22.3
	6–10	87	28.5
	11–15	79	25.9
	16 or more	71	23.3
Work schedule	Rotating	252	82.6
	Day only	41	13.4
	Night only	12	3.9

Note. EMT: Emergency Medical Technician.

**Table 2:** Descriptive statistics, internal consistency, and Pearson (upper triangle) and Spearman (lower triangle) correlations among the study variables (*n* = 305).

Variable	Mean (SD)	Range	α	Skew	Kurt	1	2	3	4
1. PTSD	27.4 (9.08)	9–45	0.89	−0.21	−0.69	–	0.611 ***	0.610 ***	0.461 ***
2. Stress	15.2 (4.36)	4–20	0.85	−0.66	−0.51	0.608 ***	–	0.812 ***	0.560 ***
3. Exhaustion	14.6 (7.37)	0–24	0.93	−0.29	−1.10	0.607 ***	0.825 ***	–	0.624 ***
4. Quiet Quitting	21.3 (7.13)	7–35	0.78	−0.01	−0.66	0.455 ***	0.577 ***	0.620 ***	–

Note. SD = standard deviation; α = Cronbach’s alpha; PTSD = post-traumatic stress disorder; Exhaustion = job-related emotional exhaustion. All correlations were significant at *** *p* < 0.001.

**Table 3:** Serial mediation analysis: total, direct, and indirect associations of PTSD symptoms with quiet quitting via perceived occupational stress and emotional exhaustion (PROCESS Model 6; 5000 bootstrap resamples; *n* = 305).

Path	B	SE	β	95% CI Lower	95% CI Upper
Total effect (c)	0.361	0.040	0.460 ***	0.282	0.440
Direct effect (c′)	0.082	0.045	0.105	−0.007	0.172
Total indirect	0.279	0.041	0.355 ***	0.199	0.364
PTSD → Stress → QQ	0.058	0.036	0.074	−0.006	0.134
PTSD → Exhaustion → QQ	0.065	0.021	0.083 ***	0.029	0.113
PTSD → Stress → Exhaustion → QQ	0.155	0.032	0.197 ***	0.091	0.217

Note. B = unstandardised coefficient; SE = standard error; β = standardised coefficient; CI = bias-corrected bootstrap confidence interval; QQ = quiet quitting. *** *p* < 0.001.

**Table 4:** Nested model comparison of the serial and single-mediator models.

Model	R^2^	Cohen’s f^2^	ΔR^2^ vs. A	*p*
A: Serial (Stress → Exhaustion)	0.421	0.727	–	–
B: Exhaustion only	0.395	0.653	0.026 *	<0.05
C: Stress only	0.341	0.517	0.080 ***	<0.001

Note. * *p* < 0.05; *** *p* < 0.001.

## Data Availability

The anonymized dataset and SPSS syntax used to produce the analyses reported in this study are available from the first author (M.T., mehmet.tatli@sbu.edu.tr) upon reasonable request by bona fide researchers affiliated with recognised academic or research institutions, subject to the terms of the ethics approval, which restricts sharing to non-commercial academic purposes. A data-sharing agreement is required. An earlier version of this manuscript was previously deposited on Research Square (https://doi.org/10.21203/rs.3.rs-9080440/v1). The present submission draws on the same participant sample but has been substantially reconceptualised, with hypotheses refined to a serial-mediation framework, the analytic approach changed to PROCESS Model 6 with bootstrap confidence intervals, and common method variance and model-comparison analyses added.

## Supplementary Materials

The following supporting information can be downloaded at: https://www.mdpi.com/article/10.3390/healthcare14152244/s1. Table S1: Covariate-adjusted serial mediation analysis controlling for gender and age (PROCESS Model 6). Table S2: Completed STROBE checklist for cross-sectional studies. Table S3: Measurement model—standardised CFA loadings, composite reliability (CR), and average variance extracted (AVE). Table S4: Discriminant validity—heterotrait–monotrait (HTMT) ratios and latent factor correlations.

## Abbreviations

PTSD: Post-Traumatic Stress Disorder; EMS: Emergency Medical Services; WPV: Workplace Violence; QQ: Quiet Quitting; PTSD-SS: PTSD-Short Scale; POSS: Perceived Occupational Stress Scale; JREES: Job-Related Emotional Exhaustion Scale; QQS: Quiet Quitting Scale; COR: Conservation of Resources; JD-R: Job Demands–Resources; CI: Confidence Interval; SE: Standard Error; VIF: Variance Inflation Factor; CMV: Common Method Variance; STROBE: Strengthening the Reporting of Observational Studies in Epidemiology.
