# Supplementary material for: Post-Traumatic Stress Symptoms, Occupational Stress, Emotional Exhaustion, and Quiet Quitting in Workplace-Violence-Exposed Turkish Prehospital Emergency Workers: A Cross-Sectional Study
Source: Healthcare (Basel). 2026 Jul 23;14(15):2244. doi: 10.3390/healthcare14152244 (PMC13465727; doi:10.3390/healthcare14152244)
Supplement: Supplementary file 1 [file healthcare-14-02244-s001.zip › healthcare-4442522-supplementary.pdf]

Supplementary Material

PTSD Symptoms, Occupational Stress, Emotional Exhaustion, and Quiet Quitting Among Turkish Prehospital Emergency Workers Exposed to Workplace Violence: A Serial Mediation Analysis

---

Table S1. Covariate-adjusted serial mediation analysis controlling for gender and age (PROCESS Model 6; 5,000 bootstrap resamples; N = 305).

Effect / Path	Relation	B	SE	95% CI [Lower, Upper]	β	t	p
**Direct and total effects**							
Total effect	PTSD to QQ	0.359	0.041	[0.279, 0.439]	.458	8.845	< .001
Direct effect	PTSD to QQ	0.089	0.045	[0.000, 0.179]	.114	1.967	.051
**Component paths**							
Path a1	PTSD to POSS	0.285	0.022	[0.241, 0.328]	.593	12.858	< .001
Path a2	PTSD to JREES	0.145	0.034	[0.078, 0.211]	.178	4.298	< .001
Path d21	POSS to JREES	1.185	0.070	[1.047, 1.324]	.701	16.864	< .001
Path b1	POSS to QQ	0.179	0.129	[-0.075, 0.432]	.109	1.388	.166
Path b2	JREES to QQ	0.454	0.076	[0.305, 0.603]	.470	5.998	< .001
**Covariates**							
Gender to POSS		0.381	0.402	[-0.411, 1.173]	.043	0.946	.345
Age to POSS		-0.754	0.345	[-1.433, -0.075]	-.099	-2.186	.030
Gender to JREES		0.378	0.492	[-0.590, 1.345]	.025	0.768	.443
Age to JREES		0.172	0.424	[-0.663, 1.006]	.013	0.405	.686
Gender to QQ		-	0.645	[-2.393,	-	-1.741	.083

Effect / Path	Relation	B	SE	95% CI [Lower, Upper]	β	t	p
		1.123		0.147]	.079		
Age to QQ		-0.917	0.556	[-2.012, 0.178]	-.074	-1.649	.100
**Indirect effects**		B	BootSE	BootCI [Lower, Upper]	β		
Total indirect	PTSD to POSS to JREES to QQ	0.270	0.041	[0.193, 0.352]	.344		
Ind1	PTSD to POSS to QQ	0.051	0.035	[-0.013, 0.128]	.065		
Ind2	PTSD to JREES to QQ	0.066	0.021	[0.029, 0.113]	.084		
Ind3 (serial)	PTSD to POSS to JREES to QQ	0.153	0.030	[0.095, 0.214]	.196		
Contrast (Ind2 vs Ind3)	Ind2 minus Ind3	-0.088	0.029	[-0.151, -0.037]	-.112		

Note. CI = confidence interval; BootSE = bootstrap standard error; bootstrap sample size = 5,000. PTSD = post-traumatic stress disorder symptoms (PTSD-SS); POSS = Perceived Occupational Stress Scale; JREES = Job-Related Emotional Exhaustion Scale; QQ = quiet quitting (Quiet Quitting Scale). Gender was coded as a binary variable; age was entered as a continuous covariate. Bootstrap confidence intervals that do not include zero indicate statistically significant indirect effects.

After adjustment for gender and age, the indirect effect through emotional exhaustion (Ind2) and the serial indirect effect through perceived occupational stress and emotional exhaustion (Ind3) remained statistically significant, whereas the single-mediator pathway through perceived occupational stress alone (Ind1) remained non-significant. The adjusted estimates were materially unchanged from the unadjusted model reported in Table 3 of the main text, indicating that the key indirect effects were robust to adjustment for these demographic covariates. A parallel sensitivity analysis entering gender and years of professional experience as covariates (not tabulated) produced the same substantive pattern.

Table S2. STROBE Statement — Checklist of items that should be included in reports of cross-sectional studies

Manuscript: healthcare-4442522 — "Post-Traumatic Stress Symptoms, Occupational Stress, Emotional Exhaustion, and Quiet Quitting in Workplace-Violence-Exposed Turkish Prehospital Emergency Workers: A Cross-Sectional Study"

**Section/Topic**	**Item No.**	**Recommendation**	**Reported in (section)**
Title and abstract	1a	Indicate the study's design with a commonly used term in the title or the abstract	Title ("A Cross-Sectional Study"); Abstract, Methods
	1b	Provide in the abstract an informative and balanced summary of what was done and what was found	Abstract (structured: Background, Methods, Results, Conclusions)
Introduction	2	Explain the scientific background and rationale for the investigation being reported	Section 1 (Introduction), paragraphs 1–4
	3	State specific objectives, including any prespecified hypotheses	Section 1, final paragraph (primary and secondary objectives); Section 2, hypotheses H1–H4
Methods	4	Present key elements of study design early in the paper	Section 2.1 (Study design)
	5	Describe the setting, locations, and relevant dates, including periods of recruitment and data collection	Section 2.2 (data collected 25 October 2025 – 7 February 2026; 112 prehospital EMS, Türkiye)
	6	Give the eligibility criteria, and the sources and methods of selection of participants	Section 2.2 (eligibility criteria i–iv; convenience sampling via online survey)
	7	Clearly define all	Sections 2.4.1–2.4.4

		outcomes, exposures, predictors, potential confounders, and effect modifiers; give diagnostic criteria, if applicable	(PTSD-SS, POSS, JREES, QQS); Section 2.5 (age and gender as covariates)
	8	For each variable of interest, give sources of data and details of methods of assessment (measurement)	Sections 2.4.1–2.4.4 (validated Turkish adaptations; scoring ranges; internal consistency in the present sample)
	9	Describe any efforts to address potential sources of bias	Section 2.5 (Harman's single-factor test for common method variance; multicollinearity diagnostics); Section 3.7 (CFA, discriminant validity); Section 4.4 (Limitations)
	10	Explain how the study size was arrived at	Section 2.2 (G*Power 3.1 calculation; Fritz & MacKinnon simulation tables for indirect effects)
	11	Explain how quantitative variables were handled in the analyses; describe which groupings were chosen and why	Sections 2.4–2.5 (continuous scale totals; no categorisation of predictors)
	12a	Describe all statistical methods, including those used to control for confounding	Section 2.5 (PROCESS Model 6, 5,000 bias-corrected bootstrap resamples; covariate-adjusted sensitivity analysis)
	12b	Describe any methods used to examine subgroups and interactions	Not applicable (no subgroup or interaction analyses were prespecified)

	12c	Explain how missing data were addressed	Section 3.1 (complete-case data; the survey required responses to all items, no item-level missingness)
	12d	If applicable, describe analytical methods taking account of sampling strategy	Section 2.2 and 4.4 (convenience sampling acknowledged; no weighting applied)
	12e	Describe any sensitivity analyses	Section 2.5 and 3.4 (age- and gender-adjusted PROCESS Model 6, Supplementary Table S1); Section 3.7 (latent-variable model)
Results	13	Report numbers of individuals at each stage of study	Section 3.1 and Figure 2 (327 accessed; 22 excluded with reasons; 305 analysed)
	14	Give characteristics of study participants and information on exposures and potential confounders	Section 3.1, Table 1 (demographic and occupational characteristics)
	15	Report numbers of outcome events or summary measures	Section 3.3, Table 2 (descriptive statistics, reliabilities, correlations)
	16a	Give unadjusted estimates and, if applicable, confounder-adjusted estimates and their precision	Sections 3.4–3.5, Table 3 (unadjusted paths with 95% bootstrap CIs); Supplementary Table S1 (covariate-adjusted)
	16b	Report category boundaries when continuous variables were categorised	Not applicable (no continuous variables were categorised)
	16c	If relevant, consider	Not applicable (no

		translating estimates of relative risk into absolute risk	relative-risk estimates)
	17	Report other analyses done — e.g., analyses of subgroups and interactions, and sensitivity analyses	Section 3.6 (nested model comparison, Table 4); Section 3.7 (four-factor CFA, Fornell–Larcker, HTMT, latent-variable SEM; Supplementary Tables S3–S4)
Discussion	18	Summarise key results with reference to study objectives	Section 4.1, first paragraph; Section 5
	19	Discuss limitations of the study, taking into account sources of potential bias or imprecision	Section 4.4 (Limitations and future directions: design, common-method bias, sampling, measurement, unmeasured confounding)
	20	Give a cautious overall interpretation of results considering objectives, limitations, multiplicity of analyses, results from similar studies, and other relevant evidence	Sections 4.1–4.3 and 5 (associations interpreted as cross-sectional statistical patterns; no causal claims)
	21	Discuss the generalisability (external validity) of the study results	Section 4.4 (fifth limitation) and Section 4.3 (Turkish prehospital context)
Other information	22	Give the source of funding and the role of the funders for the present study	Back matter, Funding statement (no external funding)

Note: Section numbers refer to the revised manuscript (v2 revision, July 2026). STROBE = Strengthening the Reporting of Observational Studies in Epidemiology.

Table S3. Measurement model — standardised CFA loadings, composite reliability (CR), and average variance extracted (AVE) (n = 305).

Construct	k	Loading range	CR	AVE	√AVE
PTSD symptoms	9	.57–.80	.90	.50	.71
Perceived occupational stress	4	.59–.83	.85	.59	.77
Emotional exhaustion	6	.70–.91	.93	.70	.83
Quiet quitting	7	.30–.80	.77	.35	.59

Table S4. Discriminant validity — HTMT ratios (below diagonal), latent factor correlations (above diagonal); √AVE on the diagonal.

	PTSD	Stress	Exhaustion	Quiet quitting
PTSD	.71	.708	.658	.540
Stress	.681	.77	.910	.700
Exhaustion	.666	.901	.83	.732
Quiet quitting	.557	.678	.738	.59

Note. Four-factor CFA fit: $\chi^2(293) = 618.4$, CFI = .930, TLI = .922, RMSEA = .060. A two-factor (stress/exhaustion) model fit significantly better than a one-factor strain model (CFI .945 vs .924; $\Delta\chi^2(1) = 52.8$, $p < .001$). Latent-variable serial SEM (CFI = .930, RMSEA = .060) standardised paths: PTSD→stress .71, stress→exhaustion .89, PTSD→exhaustion .03, stress→quiet quitting .15 (ns), exhaustion→quiet quitting .55, PTSD→quiet quitting direct .07 (ns); a single-latent-strain alternative fit slightly less well (CFI = .917). The perceived stress–emotional exhaustion pair exceeds the HTMT .90 threshold and violates the Fornell–Larcker criterion (latent r = .91 > √AVE), indicating strong overlap; all other construct pairs met discriminant-validity criteria.
